# Supplementary material for: BLMM: Parallelised computing for big linear mixed models
Source: Neuroimage. 2022 Dec 1;264:119729. doi: 10.1016/j.neuroimage.2022.119729 (PMC10985650; doi:10.1016/j.neuroimage.2022.119729)
Supplement: Supplementary Data S1 — Supplementary Raw Research Data. This is open data under the CC BY license http://creativecommons.org/licenses/by/4.0/ [file mmc1.pdf]

BLMM: Parallelised Computing for Big Linear Mixed Models Supplementary Material

Thomas Maullin-Sapey[*,1] and Thomas E. Nichols[1]

[1] Big Data Institute, Li Ka Shing Centre for Health Information and Discovery, Old Road Campus, Oxford, OX3 7LF, UK
[*]Corresponding Author: Thomas, TM, Maullin-Sapey, Thomas.Maullin-Sapey@bdi.ox.ac.uk
Submission for NeuroImage

S1 BLMM Illustrative Examples

To illustrate the notation of Sections 1.2 and 2.1.1 of the main text in practice, in this section we provide several brief mock examples of BLMM analyses. For each example, we demonstrate the notation used in the main text, describe the input that would be required by BLMM to evaluate the model and contrast this input to the syntax commonly adopted by the popular R package 'lme4'. It is emphasized that the number of subjects and observations used in the following examples are far too small to draw meaningful inference from in practice and that these examples have only been constructed for illustrative purposes. For further detailed discussion of the LMM terminology and notation employed in this work, we refer the reader to our previous work, Maullin-Sapey and Nichols [2021].

S1.1 Example (a): Longitudinal Design

The first example presented in this section consists of a "small" longitudinal group-level analysis. The analysis design contains 3 subjects, each of which has had multiple image acquisitions taken irregularly across repeated visits and has recorded observations for "sex", "age", and "BMI". Only one grouping factor for the random effects is included in the model, the factor "subject", and acquisitions for the first, second and third subjects were taken across 2, 3 and 2 visits, respectively. For this example, if the user wished to model a between-subject random intercept and a between-subject random slope for age using BLMM, the input for the matrices X, g_1 and z_1, and the full construction of Z, may appear as follows:

$$X = \begin{bmatrix} 1 & 1 & 19 & 18.7 \\ 1 & 1 & 21 & 19.2 \\ 1 & 1 & 18 & 24.8 \\ 1 & 1 & 20 & 25.1 \\ 1 & 1 & 24 & 24.6 \\ 1 & 0 & 21 & 20.9 \\ 1 & 0 & 22 & 20.1 \end{bmatrix}, \quad g_1 = \begin{bmatrix} 1 \\ 1 \\ 2 \\ 2 \\ 2 \\ 3 \\ 3 \end{bmatrix}, \quad z_1 = \begin{bmatrix} 1 & 19 \\ 1 & 21 \\ 1 & 18 \\ 1 & 20 \\ 1 & 24 \\ 1 & 21 \\ 1 & 22 \end{bmatrix}, \quad Z = \begin{bmatrix} 1 & 19 & 0 & 0 & 0 & 0 \\ 1 & 21 & 0 & 0 & 0 & 0 \\ 0 & 0 & 1 & 18 & 0 & 0 \\ 0 & 0 & 1 & 20 & 0 & 0 \\ 0 & 0 & 1 & 24 & 0 & 0 \\ 0 & 0 & 0 & 0 & 1 & 21 \\ 0 & 0 & 0 & 0 & 1 & 22 \end{bmatrix}$$

where the four columns of X correspond to an intercept, sex, age and BMI respectively, g_1 indicates which observations correspond to which subjects, and z_1 includes an intercept and age.

In terms of the notation introduced in Section 1.2.1 of the main text, the number of random factors in this model is one ($r = 1$) as there is only one factor, the factor 'subject', present in the design. The number of levels belonging the factor 'subject' is the number of subjects (e.g. $l_1 = 3$). The number of random effects which the factor 'subject' groups is two (e.g. $q_1 = 2$), as the model includes both a random intercept and a random slope for age at the subject-level. The within-subject covariance matrix, D^1, is 2×2 in dimension and contains three covariance components (the variances of the random intercept and slope on the diagonal, and the covariance between the random intercept and slope reflected off the diagonal). The random effects covariance matrix, D, is block diagonal and consists of D^1 repeated l_1 times (once per subject) along its diagonal. In this instance, for $j \in \{1,2,3\}$ the matrix $Z_{(1,j)}$, is a 7×2 dimensional matrix containing the $(2j-1)^{th}$ and $(2j)^{th}$ columns of Z (i.e. the random effects corresponding to subject j).

In this example, the input for the response vector, Y, would be a text file containing a list of the filenames of seven NIfTI BOLD response images (one for each visit and subject). During input specification, the user may also specify hypothesis tests to be conducted. For example, to perform an approximate Wald T-test testing for the non-zero effect of BMI in the above example, the user may specify T as the statistic type and $[0,0,0,1]$ as the contrast vector. For this example, the BLMM inputs file would appear as follows:

```
Y_files: Text file containing list of NIFTI images for each subject and visit
X: csv file containing X
Z:
  - f1:
      name: subject_level_random_effects
      factor: csv file containing g1
      design: csv file containing z1
outdir: /path/to/output/directory/
contrasts:
  - c1:
```

```
      name: T_contrast
      vector: [0, 0, 0, 1]
```

For comparison to the commonly adopted syntax in R, denote the ith column of X as Xi and the jth column of z_1 as z1j. An equivalent expression for the model can now be given in the syntax of lme4 as follows:

```
Y_files ~ X1 + X2 + X3 + X4 + ( z11 + z12 | g1 )
```

Note that, as X1 and z11 are both intercept columns, which are included by default in lme4, the above is equivalent to:

```
Y_files ~ X2 + X3 + X4 + ( z12 | g1 )
```

S1.2 Example (b): Longitudinal Design, Independent Random Effects

The covariance structures supported by BLMM mimic those historically supported by lmer in R. This means that the random effects covariance matrix, D, may be either diagonal or block-diagonal. These cases correspond to modelling either no correlation between random effects or 'within-factor' correlation between random effects, respectively.

In the previous example, the model contained three random effects covariance components; the variance of the subject-level random intercept, the variance of the subject-level random slope and the covariance between the two. In many practical applications, it may be desirable to assume zero correlation between the random intercept and slope. This can be achieved in BLMM by listing the factor 'subject' separately for each random effect in the inputs YAML file. The BLMM inputs YAML file for such a design would appear as follows:

```
Y_files: Text file containing list of NIFTI images for each subject and visit
X: csv file containing X
Z:
  - f1:
      name: subject_level_intercept
      factor: csv file containing g1
      design: csv file containing the first column of z1
  - f2:
      name: subject_level_age_slope
      factor: csv file containing g1
      design: csv file containing the second column of z1
outdir: /path/to/output/directory/
contrasts:
  - c1:
      name: T_contrast
      vector: [0, 0, 0, 1]
```

This example is directly equivalent to the following syntax in R's lme4:

```
Y_files ~ X1 + X2 + X3 + X4 + ( 0 + z11 | g1 ) + ( 0 + z12 | g1 )
```

In both instances, 'repeating' the factor g_1 will result in the random intercept and slope being treated independently.

Mathematically, this model may be conceptualized in one of two equivalent ways. The repeated factor 'subject' can be envisioned as two factors (e.g. $r = 2$), each of which groups one random effect; the random intercept and random slope for age, respectively ($q_1 = 1, q_2 = 1$). As there are 3 subjects, both factors contain 3 levels (e.g. $l_1 = l_2 = 3$). The "first" within-subject covariance matrix, D^1, is 1×1 in dimension and consists of only one variance component (the variance of the random intercept), and the "second" within-subject covariance matrix, D^2, is also 1×1 in dimension and consists of only one variance component (the variance of the random slope). In this instance, the random effects covariance matrix, D, is diagonal and consists of D^1 repeated 3 times along its diagonal followed by D^2 repeated 3 times along its diagonal.

Alternatively, the model may be viewed as containing only one random factor. In this instance, the model is identical to that of the previous subsection, except that D^1, which is 2×2 in this instance, contains zero-valued off-diagonal elements. As a result, when viewed in this manner, the random effects covariance matrix, D, consists of the matrix D^1 repeated 3 times along its diagonal. In both instances, the random effects covariance matrix D contains 3 copies of each random effects variance (the variances of the random slope and intercept) along its diagonal, with zero-valued off diagonal elements. As a consequence, by permuting entries of the random effects vector, b, it can be shown that these two formulations of the model are directly equivalent and will produce identical results.

S1.3 Example (c): Crossed Factors

The examples of the previous sections concern data grouped by 'subject'. However, BLMM also supports random effects with more complex grouping structures. For example, suppose that the user specified an analysis in which observations were grouped by both subject and site (location of scan). Suppose further that each subject performed a memory test and has recorded observations for "sex", "age", and "Memory_score" and the user's analysis includes a subject-level random intercept, a subject-level random slope for "Memory_score" and a site-level random intercept. Assume that the analysis design contains 4 subjects scanned at two sites where each subject had multiple image acquisitions taken irregularly across repeated visits and potentially at multiple sites.

For illustration, the matrices X, g_1 and z_1, and the full construction of Z, in this example, may appear as follows:

$$X = \begin{bmatrix} 1 & 1 & 23 & 29 \\ 1 & 1 & 31 & 43 \\ 1 & 0 & 19 & 78 \\ 1 & 1 & 20 & 23 \\ 1 & 1 & 34 & 76 \\ 1 & 0 & 21 & 54 \\ 1 & 0 & 32 & 63 \\ 1 & 1 & 24 & 88 \\ 1 & 1 & 24 & 27 \\ 1 & 0 & 29 & 45 \\ 1 & 0 & 22 & 52 \end{bmatrix}, \quad g_1 = \begin{bmatrix} 1 \\ 1 \\ 2 \\ 2 \\ 2 \\ 2 \\ 3 \\ 3 \\ 3 \\ 4 \\ 4 \end{bmatrix}, \quad z_1 = \begin{bmatrix} 1 & 29 \\ 1 & 43 \\ 1 & 78 \\ 1 & 23 \\ 1 & 76 \\ 1 & 54 \\ 1 & 63 \\ 1 & 88 \\ 1 & 27 \\ 1 & 45 \\ 1 & 52 \end{bmatrix}, \quad g_2 = \begin{bmatrix} 1 \\ 2 \\ 1 \\ 1 \\ 2 \\ 2 \\ 1 \\ 1 \\ 2 \\ 1 \\ 1 \end{bmatrix}, \quad z_2 = \begin{bmatrix} 1 \\ 1 \\ 1 \\ 1 \\ 1 \\ 1 \\ 1 \\ 1 \\ 1 \\ 1 \\ 1 \end{bmatrix},$$

$$Z = \begin{bmatrix} 1 & 29 & 0 & 0 & 0 & 0 & 0 & 0 & 1 & 0 \\ 1 & 43 & 0 & 0 & 0 & 0 & 0 & 0 & 0 & 1 \\ 0 & 0 & 1 & 78 & 0 & 0 & 0 & 0 & 1 & 0 \\ 0 & 0 & 1 & 23 & 0 & 0 & 0 & 0 & 1 & 0 \\ 0 & 0 & 1 & 76 & 0 & 0 & 0 & 0 & 0 & 1 \\ 0 & 0 & 1 & 54 & 0 & 0 & 0 & 0 & 0 & 1 \\ 0 & 0 & 0 & 0 & 1 & 63 & 0 & 0 & 1 & 0 \\ 0 & 0 & 0 & 0 & 1 & 88 & 0 & 0 & 1 & 0 \\ 0 & 0 & 0 & 0 & 1 & 27 & 0 & 0 & 0 & 1 \\ 0 & 0 & 0 & 0 & 0 & 0 & 1 & 45 & 1 & 0 \\ 0 & 0 & 0 & 0 & 0 & 0 & 1 & 52 & 1 & 0 \end{bmatrix}.$$

Here the four columns of X correspond to an intercept, sex, age and memory test score respectively, g_1 indicates which observations correspond to which subjects, z_1 includes an intercept and memory test score, g_2 indicates which observations were taken at which site and z_2 includes an intercept.

This model contains two random factors; 'subject' and 'site' (e.g. $r = 2$). The number of levels belonging to the factor 'subject' is the number of subjects (e.g. $l_1 = 4$) and the number of levels belonging to the factor 'site' is the number of sites (e.g. $l_2 = 2$). The number of random effects which the factor 'subject' groups is two (e.g. $q_1 = 2$), as the model includes both a random intercept and a random slope for memory test score at the subject-level. The number of random effects which the factor 'site' groups is one (e.g. $q_2 = 1$), as the model includes only a random intercept at the site-level. The within-subject covariance matrix, D^1, is 2×2 in dimension and contains three covariance components (the subject-level variances of the random intercept and slope and the covariance between the two). The within-site covariance matrix, D^2, is 1×1 in dimension and consists of one variance component (the variance of the site-level random intercept). The random effects covariance matrix, D, is block diagonal and consists of D^1 repeated l_1 times (once per subject) along its diagonal followed by D^2 repeated l_2 times (once per site). In this instance, for $j \in \{1,2,3,4\}$, the matrix $Z_{(1,j)}$ is the 11×2 dimensional matrix containing the $(2j-1)^{th}$ and $(2j)^{th}$ columns of Z (i.e. the random effects corresponding to subject j). For $j \in \{1,2\}$, the matrix $Z_{(2,j)}$ is the 11×1 dimensional matrix containing the $(8+j)^{th}$ column of Z (i.e. the random effects corresponding to site j).

The BLMM input YAML file for this example would appear as follows:

```
Y_files: list of bold_response images
```

```
X: csv file containing X
Z:
  - f1:
      name: subject_level_random_effects
      factor: csv file containing g1
      design: csv file containing z1
  - f2:
      name: site_level_random_effects
      factor: csv file containing g2
      design: csv file containing z2
outdir: /path/to/output/directory/
contrasts:
  - c1:
      name: T_contrast
      vector: [0, 0, 0, 1]
```

The corresponding model syntax in R's lme4 would appear as follows:

```
Y_files ~ X1 + X2 + X3 + X4 + ( z11 + z12 | g1 ) + ( z21 | g2 )
```

where Xi is the ith column of X and zij is the jth column of z_i, respectively.

S2 BLM: Parallelised Computing for Big Linear Models

In Section 2.1.2, a "product form"-based method for performing parameter estimation and inference for the Linear Model was referenced. This method served as the initial motivation for BLMM's approach to parallelized parameter estimation and inference for the Linear Mixed Model and is implemented as a sister-project to BLMM under the acronym BLM (Big Linear Models). In this appendix, we give a brief outline of the computational stages of BLM, highlighting the similarity between the approaches of BLM and BLMM.

Much like BLMM, BLM is designed to account for missingness observed as a result of mask-variability. As such, BLM assumes a model setup that is analogous to that described in Section 1.2.2 given by;

$$Y_v = M_v X \beta_v + \varepsilon_v, \qquad \varepsilon_v \sim N(0, \sigma_v^2 M_v)$$

where M_v is the $(n \times n)$-dimensional 'missingness' matrix described in Section 1.2.2 and the missing values in Y_v are encoded as zero. Mirroring the approach of Section 2.1.2, to reduce memory consumption, computation in BLM begins by evaluating the below product forms for each voxel in the analysis mask;

$$P_v = X_v' X_v, \;\; Q_v = X_v' Y_v, \;\; S_v = Y_v' Y_v,$$

where $X_v = M_v X$. Following computation of P_v, Q_v and S_v, the matrices X_v, Y_v and Z_v are redundant and discarded from memory. By employing vectorized computation in a manner similar to that described in Section 2.1.3, BLM then evaluates the ordinary least squares estimators using the product forms as follows:

$$\hat{\beta}_v = P_v^{-1} Q_v, \qquad \hat{\sigma}_v^2 = \frac{1}{n}(S_v - 2Q_v' \hat{\beta}_v + \hat{\beta}_v' P_v \hat{\beta}_v)$$

Finally, in a similar manner to that described in Section 2.1.4, BLM supports null-hypothesis testing via Wald statistics. Neglecting the voxel subscript v, Wald $T-$ and $F-$statistics are calculated using the product forms as follows:

$$T = \frac{L\hat{\beta}}{\sqrt{\hat{\sigma}_v^2 L P^{-1} L'}}, \qquad F = \frac{\hat{\beta}' L' (L P^{-1} L')^{-1} L \hat{\beta}}{\hat{\sigma}^2 \text{rank}(L)}$$

where L is a fixed and known contrast vector. For further information on the BLM toolbox, see:

https://github.com/TomMaullin/BLM

S3 Computational efficiency for product form evaluation

In Section 2.1.2, pseudocode demonstrates how BLMM evaluates the product forms in a computationally efficient manner. For clarity and conciseness, however, several practical details were neglected from Algorithm 1. Therefore, in this section, to complement the discussion of Section 2.1.2, we provide a brief overview of several practical implementation details which were neglected from Algorithm 1.

Firstly, we highlight that in Algorithm 1, several operations were conceptualized as involving the missingness matrix $M_v^{(b)}$ which has dimension $(\frac{n}{B} \times \frac{n}{B})$. Whilst the use of the matrix $M_v^{(b)}$ in this situation is theoretically justified, construction of such a matrix is computationally infeasible due to the large amount of memory required in order to store $M_v^{(b)}$ for all voxels. Here we note that, in practice, construction of the matrix $M_v^{(b)}$ is unnecessary. Instead, multiplications such as $M_v^{(b)}X_v^{(b)}$ can be evaluated incrementally by replacing rows of $X_v^{(b)}$ with zeros in an appropriate fashion.

Secondly, as a result of between-voxel design commonality (c.f. Section 1.2.2), we highlight that typically P_v, R_v and U_v will take the same values for many voxels. As such, P_v, R_v, and U_v do not need to be computed and stored separately for every voxel. Although not noted in Algorithm 1, BLMM utilises this between-voxel commonality during the product form stage of computation to reduce storage costs by storing only the unique instances of P_v, R_v and U_v. As it is often the case that there are large contiguous regions of the analysis mask over which there is little missingness, storing P_v, R_v and U_v in this manner is expected to be much more memory efficient than storing them for each voxel individually.

Thirdly, in Algorithm 1, it appears there are many “for loops” which are being serially executed across voxels. Here we highlight that the operations inside these loops utilise only conceptually simplistic operations such as matrix multiplication and addition. Consequently, in practice, these loops can be realised in a quick, efficient parallelised manner by using vectorised computation (c.f. Section 2.1.3). It must also be noted, however, that in cases when R_v and U_v are particularly large, on each node, BLMM may not be able to evaluate R_v and U_v for all voxels concurrently due to the high RAM usage that would be required. In such cases, BLMM will split $\{X_v^{(b)}\}$ and $\{Z_v^{(b)}\}$ voxel-wise into further batches and, on each node, consider each batch in serial.

Finally, it is also noted that for models which contain only one random factor, U_v becomes block diagonal. In such situations, substantial gains in computation time and memory consumption may be made via careful consideration of the structure of U_v. The benefits of this approach are most extreme when the model design contains only one random factor and one random effect. In this case, U_v is diagonal, and BLMM needs only to evaluate and store the diagonal elements of U_v. Such considerations are discussed in greater detail in the following section, Section S4.

S4 Computational efficiency for single-factor designs

This section outlines two common scenarios in which structural features of the random effects covariance and design matrices, D and Z, allow for further improvements in terms of computational efficiency in the BLMM pipeline. To illustrate how BLMM exploits the structure of D and Z to achieve improved computational performance in each of these scenarios, we shall use Equation (A.1) as an informative example throughout this section. For the remaining expressions listed in the main text and appendices, we note that similar adjustments are possible but, for brevity, will not be listed here.

The first scenario in which BLMM accounts for the structure of Z and D computationally is the setting in which the experimental design contains a single random factor which groups multiple random effects (i.e. $r = 1$ and $q_1 > 1$). In such instances, U (which is equal to $Z'Z$) and D are both block diagonal, with D consisting of only one block, D^1, which is of dimension $(q_1 \times q_1)$, repeated l_1 times along it's diagonal (i.e. $D = I_{l_1} \otimes D^1$). By noting the structure of D and U, and neglecting the subscript s for iteration number, Equation (A.1) may be rewritten in this setting as follows:

$$\begin{aligned}
X'V^{-1}X &= P - \sum_{j=1}^{l_1} R_{(j)} D^1 (I + D^1 U_{(j)})^{-1} R'_{[j]} \\
Z'V^{-1}Z &= \bigoplus_{j=1}^{l_1} \left(U_{(j)} - U_{(j)} D^1 (I_{q_1} + D^1 U_{(j)})^{-1} U_{(j)} \right) \\
Z'V^{-1}e &= T' - R'\beta - \bigoplus_{j=1}^{l_1} \left(U_{(j)} D^1 (I_{q_1} + D^1 U_{(j)})^{-1} \right) (T' - R'\beta)
\end{aligned} \tag{S1}$$

where $U_{(j)}$ and $R_{(j)}$ are shorthand for $Z'_{(1,j)}Z_{(1,j)}$ and $X'Z_{(1,j)}$ respectively, and $\oplus$ is the direct sum. Despite appearing notationally convoluted, the above expressions are much more convenient than Equation (A.1) for computational purposes. This is due to the notably smaller dimensions of the matrices involved in the right hand side of Equation (S1) in comparison to those employed in Equation (A.1).

For example, consider the dimensions of the matrices which are inverted in Equation (S1), $\{(I_{q_1} + D^1 U_{(j)})\}_{j \in \{1,\dots,l_1\}}$, and the matrix which is inverted in Equation (A.1), $(I_q + DU)$. The former matrices have dimension $(q_1 \times q_1)$ whilst the latter has dimension $(q \times q)$. To illustrate the significance of this observation, consider a setting in which 2 random effects are modelled across 100 levels (subjects or time-points, for example). In such a scenario, $q = 200$ whilst $q_1 = 2$ and, by using the above reformulation of Equation (A.1), the inversion of a matrix of dimension (200×200) can be substituted for the much faster operation of inverting 100 matrices of dimension (2×2). When applied in the mass-univariate setting, such improvements in efficiency can reduce computation time from hours to seconds.

The second scenario to which computation in the BLMM pipeline has been tailored is the setting in which the experimental design contains a single random factor that groups a single random effect (i.e. $r = 1$ and $q_1 = 1$). In this instance, the matrices D and U are diagonal, with D containing a single scalar value, d, repeated l_1 times along the diagonal. Consequently, by denoting u as the vector composed of the diagonal elements of U, Equation (A.1) can be rewritten in this setting as follows:

$$\begin{aligned}
X'V^{-1}X &= P - d(RR' \odot \text{diag}(1 \oslash (1 + du)) \\
Z'V^{-1}Z &= \text{diag}(u \odot (1 - du) \oslash (1 + du)) \\
Z'V^{-1}e &= (1 - du) \oslash (1 + du) \odot (T' - R'\beta)
\end{aligned} \tag{S2}$$

where $\odot$ and $\oslash$ represent Hadamard (element-wise) multiplication and division, respectively, and diag is the unique function which maps an arbitrary $(m \times 1)$ vector, x, to an $(m \times m)$ diagonal matrix with the elements of x listed along its diagonal. As in the previous scenario, in which the use of Equation (S1) greatly reduced the time complexity of computation, the use of Equation (S2) can also provide strong improvements in terms of computational speed. A notable example of this can be seen by contrasting the time complexity of the evaluation of $Z'V^{-1}Z$ obtained using the right-hand side of Equation (S2) against that obtained using Equation (A.1). The time complexity of the former is $O(q)$, whereas the time complexity of the latter is greater than $O(q^2)$. As a consequence, in this scenario, the matrix

$Z'V^{-1}Z$, which is utilised many times throughout the BLMM pipeline, may be evaluated in a much faster manner via the use of Equation (S2) than by use of Equation (A.1).

In each of the above scenarios, it must further be noted that, by utilising the internal structure of Z, memory consumption can also be significantly reduced. For example, when storing the product form $U = Z'Z$, only the non-zero entries of U must be recorded. This reduces the storage cost associated to U from a complexity of $O(q^2)$ to; $O(q_1 q)$ in the scenario in which U is block-diagonal, and $O(q)$ in the scenario in which U is diagonal. We note here that sparse matrix methods could theoretically also be employed to obtain further improvements in computational time and storage. However, due to the present lack of vectorised support available for sparse matrix operations, such improvements are currently infeasible in practice for mass-univariate analyses. For this reason, BLMM currently does not utilise sparse matrix methodology.

S5 Expressions for degrees of freedom estimation

In Section 2.1.4, a general overview of BLMM's approach to degrees of freedom estimation was provided, with several expressions being omitted for the purposes of concision. In this section, we list the expressions which were omitted from Section 2.1.4. To be precise, here we provide closed-form expressions for the unknown covariance matrix $\text{Var}(\hat{\eta})$, and the gradient vector $\frac{dS^2(\hat{\eta})}{d\hat{\eta}}$. To state these, we partition $\text{Var}(\hat{\eta})$ and $\frac{dS^2(\hat{\eta})}{d\hat{\eta}}$ block-wise as follows:

$$\text{Var}(\hat{\eta}) = \begin{bmatrix} \mathscr{I}_{\hat{\sigma}^2,\hat{\sigma}^2} & \mathscr{I}_{\hat{\sigma}^2,\hat{D}^1} & \cdots & \mathscr{I}_{\sigma^2,\hat{D}^r} \\ \mathscr{I}_{\hat{D}^1,\hat{\sigma}^2} & \mathscr{I}_{\hat{D}^1,\hat{D}^1} & \cdots & \mathscr{I}_{\hat{D}^1,\hat{D}^r} \\ \vdots & \vdots & \ddots & \vdots \\ \mathscr{I}_{\hat{D}^r,\hat{\sigma}^2} & \mathscr{I}_{\hat{D}^r,\hat{D}^1} & \cdots & \mathscr{I}_{\hat{D}^r,\hat{D}^r} \end{bmatrix}, \qquad \frac{dS^2(\hat{\eta})}{d\hat{\eta}} = \begin{bmatrix} \frac{dS^2(\hat{\eta})}{d\hat{\sigma}^2} \\ \frac{dS^2(\hat{\eta})}{d\text{vec}(\hat{D}^1)} \\ \vdots \\ \frac{dS^2(\hat{\eta})}{d\text{vec}(\hat{D}^r)} \end{bmatrix}.$$

where $\mathscr{I}_{A,B}$ is shorthand for the covariance matrix $\text{Cov}(\frac{dl(\hat{\theta})}{d\text{vec}(A)}, \frac{dl(\hat{\theta})}{d\text{vec}(B)})$. Given the above partition $\text{Var}(\hat{\eta})$ may now be stated block-wise as follows:

$$\mathscr{I}_{\hat{\sigma}^2,\hat{\sigma}^2} = \frac{n}{2}\hat{\sigma}^{-4},$$

$$\text{for } k \in \{1,...r\}, \qquad \mathscr{I}_{\hat{\sigma}^2,\hat{D}^k} = \frac{1}{2\hat{\sigma}^2}\text{vec}'\bigg(\sum_{j=1}^{l_k} Z'_{(k,j)}\hat{V}^{-1}Z_{(k,j)}\bigg),$$

$$\text{for } k_1,k_2 \in \{1,...r\}, \qquad \mathscr{I}_{D^{k_1},D^{k_2}} = \frac{1}{2}\sum_{j=1}^{l_{k_2}}\sum_{i=1}^{l_{k_1}}\bigg(Z'_{(k_1,i)}V^{-1}Z_{(k_2,j)} \otimes Z'_{(k_1,i)}V^{-1}Z_{(k_2,j)}\bigg),$$

and the gradient vector, $\frac{dS^2(\hat{\eta})}{d\hat{\eta}}$, may be stated block-wise as:

$$\frac{dS^2(\hat{\eta})}{d\hat{\sigma}^2} = L(X'\hat{V}^{-1}X)^{-1}L',$$

$$\text{for } k \in \{1,...r\}, \qquad \frac{dS^2(\hat{\eta})}{d\text{vec}(\hat{D}^k)} = \hat{\sigma}^2\bigg(\sum_{j=1}^{l_k}\hat{B}_{(k,j)} \otimes \hat{B}_{(k,j)}\bigg),$$

where $\hat{B}_{(k,j)}$ is given by $\hat{B}_{(k,j)} = Z'_{(k,j)}\hat{V}^{-1}X(X'\hat{V}^{-1}X)^{-1}L'$ and $\hat{V} = I_n + Z\hat{D}Z'$. For use in the BLMM pipeline, the above expressions may be reformulated to be given in terms of the product forms of Section 2.1.2 via use of Equation (A.1). The reformulated expressions are lengthy, however, and therefore will not be stated in full here. Further discussion of the above expressions can be found in our previous work, Maullin-Sapey and Nichols [2021].

S6 Random mask generation

In this section, we detail the process used to generate the random masks which were applied to the simulated data of Section 2.2. To create randomly deformed masks for the simulated analyses, the standard T1 2mm MNI152 brain mask, which is available in the FSL software package, was employed. Mask generation began by first eroding the standard T1 2mm mask by approximately three voxels. This was achieved by smoothing the binary mask with an isotropic Gaussian kernel of 4 Full Width Half Maximum (FWHM) and then re-thresholding the resultant smooth image at 0.7. The purpose of this erosion was to create a mask that was approximately the same shape and size as the standard T1 2mm MNI152 brain mask but with some voxels missing near cortical boundaries and the edge of the brain. Following this, random deformations in the size and shape of the eroded analysis mask were generated. This process was achieved by multiplying an image of $N(0,1)$ Gaussian noise by 8, adding the result to the eroded T1 2mm mask and smoothing the resultant sum with an isotropic Gaussian kernel of 10 FWHM. The final image was then re-thresholded at 0.6. This process was established empirically to ensure that the masks produced had approximately the same quantity of voxels as a standard fMRI analysis mask, with enough random deformation present to exhaustively test the missingness handling capacity of BLMM.

Using the masks generated by this process, the final analysis mask in each simulation instance (following the application of a 50% missingness threshold, c.f. Section 2.1.1) contained on average 217930.6 voxels, with a standard error of approximately 127.0 voxels across simulation instances. This meant that the final analysis mask occupied approximately 21.8% of each NIfTI volume. As noted earlier, this is, by design, similar to the 22.8% of the NIfTI volume that is occupied by the original FSL T1 2mm brain mask and may be expected to be occupied in a standard fMRI analysis. On average, in each simulation instance, approximately 87483.9 voxels had missing data (40.1% of the total number of voxels in the analysis mask), and 130446.7 voxels had full observations across all volumes generated (59.9%). We emphasize here that this extreme degree of subject-mask variability was deliberately simulated to stress test the missingness handling and time efficiency capabilities of BLMM.

S7 Mean Absolute Difference for Parameter Estimation (n=200)

Method	Missing-data voxels	Full-data voxels	All voxels
Simulation 1			
β	6.86×10^{-9}	4.75×10^{-9}	5.45×10^{-9}
	(1.32×10^{-11})	(9.14×10^{-12})	(1.02×10^{-11})
σ^2	1.43×10^{-9}	1.06×10^{-9}	1.18×10^{-9}
	(3.10×10^{-12})	(2.21×10^{-12})	(2.46×10^{-12})
D	8.26×10^{-6}	5.20×10^{-6}	6.20×10^{-6}
	(1.67×10^{-8})	(1.10×10^{-8})	(1.24×10^{-8})
Simulation 2			
β	4.39×10^{-5}	2.34×10^{-5}	3.05×10^{-5}
	(2.05×10^{-7})	(1.43×10^{-7})	(1.62×10^{-7})
σ^2	6.12×10^{-6}	3.33×10^{-6}	4.29×10^{-6}
	(2.44×10^{-8})	(1.70×10^{-8})	(1.92×10^{-8})
D	2.54×10^{-4}	1.40×10^{-4}	1.79×10^{-4}
	(1.24×10^{-5})	(8.59×10^{-6})	(9.78×10^{-6})
Simulation 3			
β	1.70×10^{-5}	8.01×10^{-6}	1.11×10^{-5}
	(1.13×10^{-7})	(7.53×10^{-8})	(8.73×10^{-8})
σ^2	1.27×10^{-6}	5.44×10^{-7}	7.95×10^{-7}
	(7.13×10^{-9})	(4.11×10^{-9})	(5.09×10^{-9})
D	6.02×10^{-3}	2.93×10^{-3}	3.99×10^{-3}
	(4.10×10^{-5})	(2.72×10^{-5})	(3.17×10^{-5})

Table S1: Mean absolute difference in the estimates produced by lmer and BLMM for β, σ^2 and D, averaged across voxels and simulation instances. Each reported average is an image-wide mean, taken across approximately $218,000$ voxels, further averaged across 1000 simulation instances. In each simulation instance, the model employed included 200 observations generated according to the methods outlined in Section 2.2. Empirical standard errors, taken across simulation instances, are also given in brackets underneath each entry in the table.

S8 Mean Absolute Difference for Parameter Estimation (n=500)

Method	Missing-data voxels	Full-data voxels	All voxels
Simulation 1			
β	1.54×10^{-9}	1.09×10^{-9}	1.26×10^{-9}
	(1.96×10^{-12})	(1.52×10^{-12})	(1.60×10^{-12})
σ^2	3.48×10^{-10}	2.54×10^{-10}	2.89×10^{-10}
	(2.51×10^{-13})	(1.69×10^{-13})	(1.78×10^{-13})
D	1.13×10^{-6}	6.64×10^{-7}	8.40×10^{-7}
	(1.26×10^{-9})	(8.30×10^{-10})	(9.06×10^{-10})
Simulation 2			
β	2.00×10^{-6}	6.63×10^{-7}	1.17×10^{-6}
	(1.13×10^{-8})	(5.62×10^{-9})	(7.60×10^{-9})
σ^2	2.61×10^{-7}	8.41×10^{-8}	1.51×10^{-7}
	(1.00×10^{-9})	(4.19×10^{-10})	(6.15×10^{-10})
D	1.35×10^{-3}	4.84×10^{-4}	8.13×10^{-4}
	(7.24×10^{-6})	(3.81×10^{-6})	(4.98×10^{-6})
Simulation 3			
β	8.10×10^{-7}	3.02×10^{-7}	4.94×10^{-7}
	(5.60×10^{-9})	(2.55×10^{-9})	(3.60×10^{-9})
σ^2	3.53×10^{-8}	1.00×10^{-8}	1.96×10^{-8}
	(1.45×10^{-10})	(3.60×10^{-11})	(7.30×10^{-11})
D	2.76×10^{-4}	9.67×10^{-5}	1.64×10^{-4}
	(2.06×10^{-6})	(9.58×10^{-7})	(1.35×10^{-6})

Table S2: Mean absolute difference in the estimates produced by lmer and BLMM for β, σ^2 and D, averaged across voxels and simulation instances. Each reported average is an image-wide mean, taken across approximately $218,000$ voxels, further averaged across 1000 simulation instances. In each simulation instance, the model employed included 500 observations generated according to the methods outlined in Section 2.2. Empirical standard errors, taken across simulation instances, are also given in brackets underneath each entry in the table.

S9 Mean Absolute Difference for Parameter Estimation (n=1000)

Method	Missing-data voxels	Full-data voxels	All voxels
Simulation 1			
β	6.33×10^{-10}	4.19×10^{-10}	5.05×10^{-10}
	(7.66×10^{-13})	(5.85×10^{-13})	(6.20×10^{-13})
σ^2	1.57×10^{-10}	1.10×10^{-10}	1.29×10^{-10}
	(8.10×10^{-14})	(5.84×10^{-14})	(5.15×10^{-14})
D	4.96×10^{-7}	2.49×10^{-7}	3.48×10^{-7}
	(5.66×10^{-10})	(3.38×10^{-10})	(3.91×10^{-10})
Simulation 2			
β	6.54×10^{-8}	1.20×10^{-8}	3.36×10^{-8}
	(2.95×10^{-10})	(4.35×10^{-11})	(1.33×10^{-10})
σ^2	6.71×10^{-9}	1.24×10^{-9}	3.46×10^{-9}
	(1.55×10^{-11})	(1.45×10^{-12})	(5.93×10^{-12})
D	6.25×10^{-5}	1.65×10^{-5}	3.51×10^{-5}
	(3.32×10^{-7})	(9.35×10^{-8})	(1.80×10^{-7})
Simulation 3			
β	5.96×10^{-8}	2.66×10^{-8}	3.99×10^{-8}
	(3.10×10^{-10})	(1.32×10^{-10})	(1.71×10^{-10})
σ^2	5.80×10^{-9}	3.78×10^{-9}	4.60×10^{-9}
	(1.29×10^{-11})	(6.80×10^{-12})	(7.17×10^{-12})
D	2.97×10^{-5}	2.07×10^{-5}	2.44×10^{-5}
	(1.02×10^{-7})	(3.29×10^{-8})	(5.74×10^{-8})

Table S3: Mean absolute difference in the estimates produced by lmer and BLMM for β, σ^2 and D, averaged across voxels and simulation instances. Each reported average is an image-wide mean, taken across approximately $218,000$ voxels, further averaged across 1000 simulation instances. In each simulation instance, the model employed included 1000 observations generated according to the methods outlined in Section 2.2. Empirical standard errors, taken across simulation instances, are also given in brackets underneath each entry in the table.

S10 Mean Absolute Difference for Maximized ReML Criteria

Method	Missing-data voxels	Full-data voxels	All voxels
Simulation 1			
$n = 200$	8.72×10^{-10}	6.16×10^{-10}	7.02×10^{-10}
	(2.98×10^{-12})	(2.23×10^{-12})	(2.21×10^{-12})
$n = 500$	6.24×10^{-10}	7.36×10^{-10}	6.95×10^{-10}
	(1.87×10^{-11})	(1.82×10^{-11})	(1.32×10^{-11})
$n = 1000$	1.11×10^{-9}	8.23×10^{-10}	9.36×10^{-10}
	(4.92×10^{-11})	(6.02×10^{-11})	(4.04×10^{-11})
Simulation 2			
$n = 200$	4.25×10^{-3}	2.42×10^{-3}	3.05×10^{-3}
	(4.14×10^{-5})	(3.04×10^{-5})	(3.39×10^{-5})
$n = 500$	2.03×10^{-4}	6.96×10^{-5}	1.20×10^{-4}
	(2.43×10^{-6})	(1.28×10^{-6})	(1.63×10^{-6})
$n = 1000$	1.02×10^{-5}	2.46×10^{-6}	5.58×10^{-6}
	(3.99×10^{-7})	(2.34×10^{-7})	(2.12×10^{-7})
Simulation 3			
$n = 200$	1.11×10^{-3}	6.72×10^{-4}	8.23×10^{-4}
	(1.29×10^{-5})	(9.51×10^{-6})	(1.05×10^{-5})
$n = 500$	2.55×10^{-4}	1.75×10^{-4}	2.05×10^{-4}
	(2.61×10^{-6})	(1.87×10^{-6})	(1.74×10^{-6})
$n = 1000$	5.78×10^{-5}	3.43×10^{-5}	4.38×10^{-5}
	(1.22×10^{-6})	(9.44×10^{-7})	(7.58×10^{-7})

Table S4: Mean absolute difference in the maximized ReML criteria produced by lmer and BLMM, averaged across voxels and simulation instances. Each reported average is an image-wide mean, taken across approximately $218,000$ voxels, further averaged across 1000 simulation instances. The number of observations present in the model used for each simulation setting is displayed on the left. All observations were generated according to the methods outlined in Section 2.2. Empirical standard errors, taken across simulation instances, are also given in brackets underneath each entry in the table.

S11 Real Data Analysis Secondary Contrasts

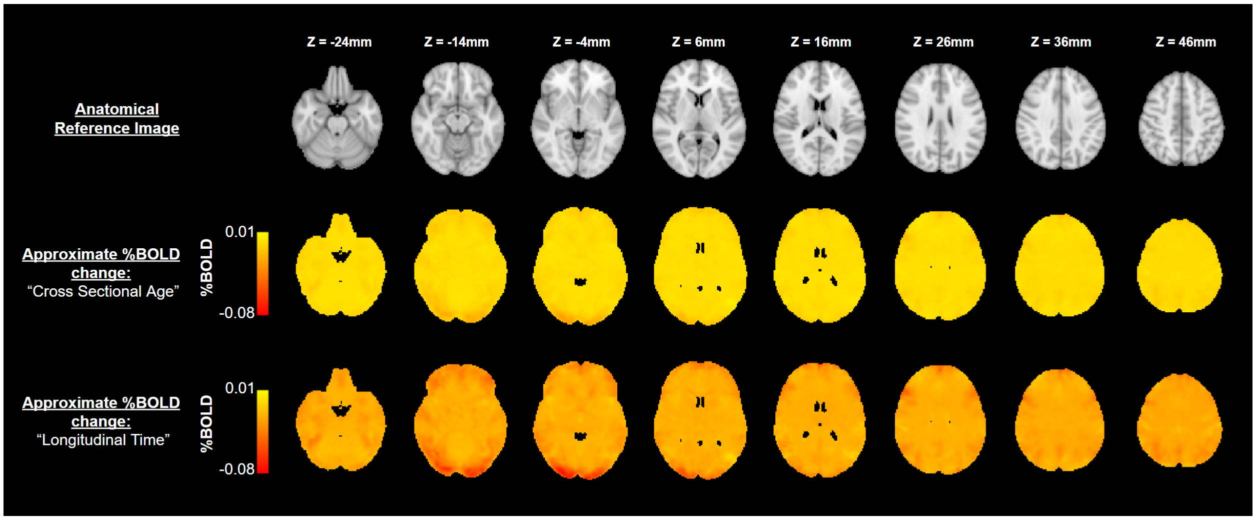

Figure S1: First row: The MNI152 2mm anatomical template, for reference. Second row: Effect estimates for the "Cross-Sectional Age" contrast, derived from model 2. Third row: Effect estimates for the "Longitudinal Time" contrast, derived from model 2. For both rows, no voxels were found to be Bonferroni-significant at the 5% level. It should be noted that the clipping ranges for %BOLD contrast shown here are extremely small in comparison to Fig. 4 of the main text. It is safe to conclude that no evidence was observed for the effect of either age covariate on the "faces vs shapes" task.

References

Thomas Maullin-Sapey and Thomas E. Nichols. Fisher scoring for crossed factor linear mixed models. *Statistics and Computing*, 31(5):53, Jul 2021. ISSN 1573-1375. doi: 10.1007/s11222-021-10026-6. URL https://doi.org/10.1007/s11222-021-10026-6.
